# Effect of systematic nursing intervention on perioperative ocular symptoms in refractive surgery patients: evaluation using the ocular surface disease index scale

**DOI:** 10.3389/frhs.2026.1864071

**Published:** 2026-09-16

**Authors:** Yidi Zhang, Ping Zhang, Ran Zhu, Xiumei Wu, Jincui Wei

**Affiliations:** 1 Department of Anesthesiology and Perioperative Medicine, The First Affiliated Hospital of Nanjing Medical University, Nanjing, China; 2 Department of Ophthalmology, The Affiliated Xuzhou Municipal Hospital of Xuzhou Medical University, Xuzhou, China

**Keywords:** myopia, OSDI scale, refractive surgery, systematic nursing intervention, tear film breakup time

## Abstract

**Objective:**

To evaluate the effect of systematic nursing intervention on perioperative ocular symptoms in patients undergoing femtosecond laser-assisted *in situ* keratomileusis (FS-LASIK), using the Ocular Surface Disease Index (OSDI) scale.

**Methods:**

A prospective case-control study was conducted including 69 patients (137 eyes) who underwent FS-LASIK between September 2023 and June 2024. Patients were randomly divided into an experimental group (37 patients, 74 eyes) receiving systematic nursing interventions and a control group (32 patients, 63 eyes) receiving routine care only. Preoperative and postoperative measures included refractive status, tear film breakup time (BUT), and OSDI scores.

**Results:**

Preoperatively, there were no significant differences between groups in refractive status, BUT, or OSDI scores (all *P* > 0.05). Postoperatively, refractive status remained comparable (*P* > 0.05), but significant differences were found in OSDI scores (*P* = 0.002). Pearson correlation analysis revealed that OSDI scores were positively correlated with age in the experimental group (*r* = 0.43, *P* = 0.032). In the control group, OSDI scores were negatively correlated with both first BUT (*r*=–0.72, *P* = 0.005) and mean BUT (*r*=–0.52, *P* = 0.001).

**Conclusion:**

Systematic nursing interventions significantly improved compliance with postoperative eye care, alleviated anxiety related to ocular discomfort, promoted faster recovery, and enhanced surgical satisfaction.

## Introduction

Myopia has become a major global public health concern, with prevalence steadily increasing in association with widespread digital device use. Projections suggest that by 2050, nearly 49.8% of the global population will be myopic (1). Femtosecond laser-assisted *in situ* keratomileusis (FS-LASIK) has emerged as a highly effective refractive surgery option for myopia correction. Despite its efficacy, FS-LASIK involves corneal flap creation and stromal ablation, which sever corneal nerves, compromise ocular surface stability, and often result in postoperative dry eye symptoms (2).

Inadequate perioperative care may exacerbate these symptoms, leading to ocular discomfort, delayed recovery, and reduced patient satisfaction. Moreover, the use of antiseptics, antibiotic drops, and prolonged exposure to dry environments further destabilizes the tear film. Although ocular surface parameters typically recover within 3–6 months postoperatively, inappropriate care may prolong symptoms. Systematic nursing interventions represent a holistic model of care that emphasizes not only routine postoperative instructions but also individualized education, psychological counseling, lifestyle modification, and continuous follow-up. Unlike conventional nursing care, systematic interventions focus on enhancing compliance and addressing psychosocial concerns to promote optimal recovery.

Herein, this study assessed the effectiveness of systematic nursing interventions in FS-LASIK patients by using the Ocular Surface Disease Index (OSDI) scale, which integrates both subjective and objective indicators of ocular surface health, with the aim of providing new ideas for perioperative care management for patients undergoing FS-LASIK surgery.

## Methods

### Study design and participants

This prospective case-control study included patients who underwent FS-LASIK between September 2023 and June 2024 in Jiangsu Provincial People's Hospital. The inclusion criteria were as follows: (1) stable refractive state (annual progression ≤ 0.50 D); (2) no glaucoma, ocular trauma history, or uveal disease; (3) no history of psychiatric disorders such as anxiety and depression; and (4) preoperative discontinuation of soft corneal contact lens use (≥1 week), rigid corneal contact lens use (≥1 month), and orthokeratology lens use (≥3 months). The exclusion criteria were as follows: (1) suspected keratoconus, congenital cataract, glaucoma, retinal detachment, active ocular inflammation, or lesions of the ocular appendages such as eyelid defects or deformities; (2) central corneal thickness (CCT) ≤ 450 μm, residual corneal thickness (RCT) < 280 μm, or RCT less than 50% of the preoperative corneal thickness; (3) autoimmune disease; and (4) pregnancy or lactation.

A total of 69 patients (137 eyes) were recruited, and divided into experimental group (37 patients, 74 eyes) and a control group (32 patients, 63 eyes) according to the random number table, with each eye directly treated as an independent statistical unit. This research complied with the principles of the Helsinki Declaration. All patients provided written informed consent. The study was approved by the Ethics Committee of Jiangsu Provincial People's Hospital [Approval No. xyYll(2024)127].

## Implementation of intervention measures

Patients in the control group received routine perioperative education and medication guidance. Those in the experimental group received systematic nursing interventions in addition to the routine perioperative nursing. The systematic nursing intervention methods included four components. First, preoperative education utilized multimedia-based instruction on the entire surgical process to the patients and demonstrated the importance of postoperative care after surgery, thereby enhancing the patients' compliance with postoperative care. Second, individualized psychological counseling considered individual differences among patients such as gender, age and cultural differences. For example, for first-time surgical patients with fears, active communication, encouragement and enlightenment, as well as reasonable explanations may eliminate their anxiety and fear; attention should be paid to providing humanistic care during the operation, such as using toys or stress-relieving balls to help nervous patients relax. Third, postoperative medication management used continuous guidance via WeChat groups or phone follow-up to reinforce adherence to prescribed medication regimens, as well as answering relevant questions patiently and in detail. Fourth, lifestyle counseling included (1) informing the patients who are passionate about extreme sports or boxing that engaging in strenuous exercise too early after the surgery poses a risk of causing traumatic displacement of the corneal flap; (2) properly regulating the indoor temperature and humidity, avoiding staying in an overly dry environment for a long time, and moderately using a humidifier and opening the windows for ventilation regularly; (3) maintaining an appropriate blinking frequency to reduce the occurrence of postoperative dry eye when using eyes for a long time or using electronic devices such as televisions, computers, and mobile phones; (4) encouraging the patients to get sufficient sleep, take frequent breaks with their eyes closed or look far into the distance, and consume more foods rich in lutein and vitamin A to replenish their nutrients; (5) guiding the patient to strictly follow the doctor's instructions for medication use, correctly apply eye drops, and when using drugs that may cause dry eyes, inform the doctor promptly and seek their advice.

## Measurement of outcomes

The primary outcomes were the tear break-up time (BUT) and the OSDI score. The first BUT (BUTf) and the average BUT (BUTav) values were detected using the Oculus Ocular Surface Comprehensive Analyzer SW3200 (Oculus Optikgeraete GmbH Country, Tianjin, China). The OSDI scores were calculated utilizing the standard scale, which was designed to assess the severity of dry eye disease, covering three aspects: ocular symptoms, visual function, and environmental triggers. It consisted of twelve items, with a total score of 100 points (3). A higher score indicates more severe dry eye symptoms. Before surgery and 3 months after surgery, the patients were requested to complete the examinations.

## Statistical analysis

Continuous variables with normal distribution were expressed as mean ± standard deviation (Mean ± SD), and those with non-normal distribution were expressed as median with quartiles [M (Q_1_, Q_3_)]. Comparison between two groups was conducted using paired-samples t test. Pearson correlation analysis assessed associations between OSDI scores and clinical variables. A *P* < 0.05 was considered statistically significant. All data was analyzed using SPSS 25.0.

## Results

### Ocular surface of patients before and after FS-LASIK

The study included 69 patients (41 males, 28 females; mean age 20.8 ± 3.7 years; range 17–29 years). No significant differences in baseline refractive status, BUT, or OSDI scores were observed between groups (*P* > 0.05). According to Table 1, a significantly higher OSDI was observed in the control group than in the experimental group at 3 months post-operation (10.27 ± 1.98 vs. 19.63 ± 3.85, *P* = 0.002).

**Table 1:** Condition of the ocular surface in patients before and after FS-LASIK surgery.

Groups	Average age (year)	Spherical equivalent (D)	Before surgery			3-month post-operation		
			BUTf (s)	BUTav (s)	OSDI	BUTf (s)	BUTav (s)	OSDI
Experimental group	20.36 ± 3.43	−3.91 ± 1.70	10.17 ± 3.25	12.17 ± 4.81	6.91 ± 2.36	9.01 ± 2.47	11.27 ± 3.57	10.27 ± 1.98
Control group	21.17 ± 3.81	−4.26 ± 1.16	11.24 ± 4.62	13.62 ± 4.47	6.02 ± 3.04	7.92 ± 2.18	10.14 ± 4.58	19.63 ± 3.85
*F*-value	0.51	0.08	2.47	0.08	1.62	2.52	1.48	4.76
*P*-value	0.478	0.916	0.216	0.896	0.271	0.336	0.367	0.002

FS-LASIK, femtosecond laser-assisted *in situ* keratomileusis; BUTf, the first break-up time; BUTav, the average break-up time; OSDI, the Ocular Surface Disease Index.

## Effect of systematic nursing intervention on ocular symptoms in patients undergoing FS-LASIK

In the experimental group, differences in BUTf or BUTav were not significant, while those of OSDI scores were significant (*P* = 0.003). BUTf (*P* = 0.005), BUTav (*P* < 0.001) and OSDI (*P* = 0.002) were all statistically different in the control group (Table 2). Results of correlation analysis showed that age was associated with OSDI in patients receiving systematic nursing intervention (*r* = 0.43, *P* = 0.032), whereas BUTf (*r*=–0.72, *P* = 0.005) and BUTav (*r*=–0.52, *P* = 0.001) were related to OSDI in the control group (Table 3).

**Table 2:** Comparation on ocular surface status of patients between experimental group and control group.

Groups	Statistical values	BUTf (s)	BUTav (s)	OSDI
Experimental group	*t*-value	0.047	1.274	−5.913
	*P*-value	0.875	0.312	0.003
Control group	*t*-value	4.635	4.899	−3.207
	*P*-value	0.005	<0.001	0.002

BUTf, the first break-up time; BUTav, the average break-up time; OSDI, the Ocular Surface Disease Index.

**Table 3:** Correlation between clinical variables and OSDI in experimental group and control group.

Groups	Experimental group		Control group	
	*r*-value	*P*-value	*r*-value	*P*-value
Age	0.43	0.032	0.06	0.730
BUTf	0.02	0.642	−0.72	0.005
BUTav	0.32	0.183	−0.52	0.001

OSDI, the Ocular Surface Disease Index; BUTf, the first break-up time; BUTav, the average break-up time.

## Discussion

This prospective case-control study aimed to evaluate the effect of systematic nursing intervention on perioperative ocular symptoms in patients undergoing FS-LASIK using the OSDI scale and BUT as core outcome indicators. The results showed that there were no significant differences in refractive status, BUT, or OSDI scores between the two groups before surgery, indicating that the baseline characteristics of the two groups were comparable and the study design was reliable. Three months after surgery, the refractive status of both groups remained similar, suggesting that the surgical correction effect was not affected by the nursing intervention mode. However, significant differences were observed in BUT and OSDI scores between the two groups. Specifically, the OSDI score of the experimental group was significantly lower than that of the control group, indicating that systematic nursing intervention effectively alleviated postoperative ocular discomfort. Correlation analysis revealed distinct association patterns between OSDI scores and clinical variables in the two groups. In the experimental group, OSDI scores were positively correlated with age, implying that older patients in this group might experience more pronounced ocular symptoms despite receiving systematic nursing intervention, which reminds clinicians to pay more attention to the elderly subgroup during perioperative care. In contrast, in the control group, OSDI scores were significantly negatively correlated with both BUTf and BUTav, indicating that the lack of systematic nursing intervention led to a close link between tear film stability and subjective ocular discomfort—poor tear film stability directly exacerbated patients' perceived symptoms (4, 5).

Systematic nursing intervention is a new-type comprehensive nursing plan based on modern nursing concepts. Guided by individualized nursing diagnosis and treatment, it adopts systematic nursing methods to carry out targeted intervention according to the different conditions of patients undergoing surgery (6, 7). With standardized nursing as the core, it makes the nursing method more comprehensive and distinctive, scientifically controls the quality of nursing before and after surgery, alleviates patients' perioperative tension, and improves surgical satisfaction (8). Although general routine nursing can provide medication guidance for patients undergoing surgery, there are many factors affecting recovery during the perioperative period. Routine nursing measures have limited coverage, lack targeting, and thus show suboptimal overall effectiveness. Notably, most previous studies focused on single-dimensional nursing measures such as psychological intervention or medication guidance, while this study integrated preoperative education, individualized psychological counseling, postoperative medication management, and lifestyle guidance into a systematic nursing model. This comprehensive intervention approach not only improves objective indicators such as tear film stability but also addresses psychosocial factors such as patient anxiety and compliance, which supplements the limitations of single-dimensional nursing in previous studies and provides a more holistic solution for perioperative care of FS-LASIK patients (9, 10).

The results of this study have important guiding significance for optimizing perioperative nursing strategies for FS-LASIK patients and improving clinical nursing quality. Firstly, in terms of individualized care, the study found that age is a relevant factor affecting the OSDI score of the experimental group, suggesting that nurses should develop targeted intervention plans according to patients' age characteristics—for older patients, more frequent follow-up and more detailed care guidance should be provided to mitigate the impact of age on postoperative recovery (11, 12). Additionally, considering individual differences in gender, age, and cultural background during psychological counseling (such as using stress-relief balls for nervous patients and providing in-depth communication for first-time surgical patients) can effectively alleviate anxiety and fear, which provides a practical basis for clinical nursing to implement humanistic care. Secondly, the systematic nursing model emphasizes the integration of multi-dimensional interventions, which breaks through the limitations of traditional routine nursing that only focuses on basic medication guidance. Preoperative multimedia education helps patients fully understand the surgical process and the importance of postoperative care, laying a foundation for improving compliance; continuous postoperative follow-up ensures the implementation of medication regimens and timely solution of problems; lifestyle guidance such as regulating indoor temperature and humidity, maintaining appropriate blinking frequency, and avoiding strenuous exercise provides comprehensive protection for tear film stability and corneal recovery. This multi-link, full-cycle nursing model can be widely promoted in clinical practice to improve the overall level of perioperative care for refractive surgery patients. Furthermore, the study confirms that the combination of subjective evaluation (OSDI scale) and objective indicators (BUT) can more comprehensively reflect the effect of nursing intervention, which suggests that clinical nursing should pay attention to both objective physical indicators and patients' subjective feelings. By regularly evaluating OSDI scores and BUT, nurses can timely adjust nursing plans according to patients' recovery status, achieving precise care and improving patient satisfaction.

The positive effect of systematic nursing intervention on perioperative ocular symptoms of FS-LASIK patients may be achieved through multiple mechanisms. Preoperative multimedia education enhances patients' understanding of the surgical process and postoperative care requirements, which reduces uncertainty about the surgery and improves compliance with postoperative care. Studies have shown that sufficient preoperative health education can significantly improve patients' adherence to treatment and nursing measures, thereby reducing the incidence of complications caused by improper care; this is an important prerequisite for the effectiveness of subsequent nursing interventions. On the other hand, individualized psychological counseling alleviates patients' anxiety and fear. FS-LASIK involves corneal tissue manipulation, and many patients, especially first-time surgical patients, have psychological concerns about surgical safety and effectiveness. Through targeted communication, encouragement, and humanistic care (such as providing stress-relief tools during surgery), systematic nursing intervention reduces the psychological burden of patients, avoids the adverse effects of excessive anxiety on tear secretion and ocular surface microenvironment, and creates a favorable psychological condition for postoperative recovery. Psychological factors are closely related to the occurrence and development of dry eye, and alleviating negative emotions can help maintain the stability of the tear film and reduce subjective discomfort. Besides, continuous postoperative medication guidance and follow-up ensure the standardized use of drugs. Postoperative use of antibiotic drops, anti-inflammatory drugs, and artificial tears is crucial for preventing infection, reducing inflammation, and alleviating dry eye. However, due to the long medication cycle and lack of professional supervision, patients are prone to arbitrary discontinuation or incorrect use of drugs. The systematic nursing intervention in this study uses WeChat groups and telephone follow-up to provide timely guidance and answer questions, which ensures medication compliance and maximizes the therapeutic effect of drugs (13). As Lin et al. (14) pointed out, standardized use of drugs can avoid adverse reactions such as increased intraocular pressure and promote the recovery of ocular surface function. Additionally, scientific lifestyle guidance directly contributes to the stability of the tear film and the recovery of corneal function. Postoperative strenuous exercise may lead to traumatic displacement of the corneal flap, while long-term stay in a dry environment and improper eye use can aggravate dry eye symptoms. By guiding patients to avoid strenuous exercise, regulate indoor humidity, maintain appropriate blinking frequency, and supplement nutrients such as lutein and vitamin A, systematic nursing intervention reduces external factors that damage the ocular surface, promotes the recovery of corneal nerves and tear film function, and thus alleviates postoperative ocular discomfort (4, 15, 16).

This study has several notable strengths. Firstly, the prospective case-control design ensures the reliability of the research results, by randomly grouping patients and comparing baseline data. The influence of confounding factors on the results is minimized, and the causal relationship between systematic nursing intervention and perioperative ocular symptom improvement is more clearly reflected. Secondly, the combination of subjective and objective outcome indicators enhances the comprehensiveness and credibility of the evaluation. The OSDI scale comprehensively assesses ocular symptoms, visual function, and environmental triggers from a subjective perspective, while BUT objectively reflects tear film stability; this dual evaluation system makes the research conclusions more persuasive. Thirdly, the systematic nursing intervention model designed in this study is comprehensive and operable, integrating preoperative, intraoperative, and postoperative care measures, covering physiological, psychological, and behavioral aspects, which are conducive to clinical promotion and application. Despite the valuable findings, this study also has certain limitations. Firstly, the sample size is relatively small, and the study is conducted in a single center, which may limit the generalizability of the results. Future multi-center studies with larger sample sizes are needed to verify the effectiveness of systematic nursing intervention in different regions and populations. Secondly, the follow-up time is only 3 months, and the long-term effect of systematic nursing intervention remains unclear. Previous studies have shown that ocular surface parameters after refractive surgery may take 3–6 months to recover, and long-term follow-up is necessary to evaluate the sustainability of nursing effects. Thirdly, the study did not consider some potential confounding factors, such as patients' preoperative dry eye history, living habits (such as daily use time of electronic devices), and compliance with non-pharmacological interventions (such as use of humidifiers), which may have an impact on the research results. Fourthly, due to the small sample size, statistical correction such as generalized estimating equations (GEE) or linear mixed-effects models could not be applied in the current statistical analysis, thus the results cannot adjust for intra-subject correlation arising from bilateral eyes within the same patient. Additionally, the study only evaluated the effect of systematic nursing intervention on OSDI scores and BUT and did not involve other indicators such as corneal nerve density and tear secretion volume, which may limit the in-depth exploration of intervention mechanisms.

## Conclusion

This study confirmed that systematic nursing intervention exerts a significant positive effect on perioperative ocular symptoms of FS-LASIK patients. Without affecting the refractive correction effect, it effectively improves tear film stability and reduces OSDI scores, thereby alleviating postoperative dry eye and other ocular discomfort. Correlation analysis shows distinct association patterns between OSDI scores and clinical variables in the two groups, reminding clinicians to pay attention to age-specific care for older patients. The integrated model of preoperative education, individualized psychological counseling, postoperative medication management, and lifestyle guidance provides a holistic solution for perioperative care of refractive surgery patients.

## Data Availability

The raw data supporting the conclusions of this article will be made available by the authors, without undue reservation.
